# An Investigation of Generalized Differential Evolution Metaheuristic for Multiobjective Optimal Crop-Mix Planning Decision

**DOI:** 10.1155/2014/258749

**Published:** 2014-04-23

**Authors:** Oluwole Adekanmbi, Oludayo Olugbara, Josiah Adeyemo

**Affiliations:** ^1^Department of Information Technology, Durban University of Technology, P.O. Box 1334, Durban 4000, South Africa; ^2^Department of Civil Engineering and Surveying, Durban University of Technology, P.O. Box 1334, Durban 4000, South Africa

## Abstract

This paper presents an annual multiobjective crop-mix planning as a problem of concurrent maximization of net profit and maximization of crop production to determine an optimal cropping pattern. The optimal crop production in a particular planting season is a crucial decision making task from the perspectives of economic management and sustainable agriculture. A multiobjective optimal crop-mix problem is formulated and solved using the generalized differential evolution 3 (GDE3) metaheuristic to generate a globally optimal solution. The performance of the GDE3 metaheuristic is investigated by comparing its results with the results obtained using epsilon constrained and nondominated sorting genetic algorithms—being two representatives of state-of-the-art in evolutionary optimization. The performance metrics of additive epsilon, generational distance, inverted generational distance, and spacing are considered to establish the comparability. In addition, a graphical comparison with respect to the true Pareto front for the multiobjective optimal crop-mix planning problem is presented. Empirical results generally show GDE3 to be a viable alternative tool for solving a multiobjective optimal crop-mix planning problem.

## 1. Introduction

The overarching objective of this work is to investigate the performance of the generalized differential evolution metaheuristic for multiobjective optimal crop-mix planning decision in the agricultural domain. The purpose of agricultural crop planning decision is generally to guarantee sufficient food resources for the human population, which is increasingly growing at a fast rate. In addition, the global demand for food items is growing at an accelerated rate. However, most of the available techniques for expanding agricultural systems have a serious long-term implication for the human environment [1]. The impact of increasing crop demand definitely depends heavily on the development of global agriculture. The needed development of the agricultural farming systems is directed toward achieving a great technology improvement. This should meet the year 2050 crop demand vision with much lower environmental impact. The impact of doubling the global crop production will depend on how increased production is achieved [2].

The intensification of agricultural practices such as clearing land for massive crop production, achieving higher yields through increased agricultural inputs, and promoting innovations through the application of information communication technology could improve crop production and agricultural value chain [3]. The increasing growth of human population across the world has called for sustainable growth in agricultural products—so as to meet the primary needs of the human population [4]. One copious strategy of sustainable agriculture is crop-mix—sometimes called mixed cropping [5]. The formulation of multiobjective optimal crop-mix planning problem as presented in this paper simplifies the task of crop planning in agriculture setting that is generally aimed at maximizing returns from the meager resources available to farmers [6].

The current work explores an approach based on evolutionary metaheuristics to solve a multiobjective optimal crop-mix optimization problem. This could suggest an effective tool to support farmers in optimal crop planning decision making. There are numerous reasons for using evolutionary metaheuristics to solve optimization problems. One of such reasons is that evolutionary metaheuristics need little problem specific knowledge and can be applied to a broad range of problem types [7]. The evolutionary metaheuristics required the target objective function for a given problem to be optimized, but additional problem specific knowledge can be easily brought into metaheuristics to improve their performances [8]. In addition, metaheuristics require no derivative information; they are robust, flexible, and relatively simple to implement [9].

Previous studies on crop planning have used single and multiobjective optimization models, including linear programming [10–13], dynamic programming [14], and evolutionary metaheuristics [15–18] to solve diverse formulations of crop planning problem. The variety of optimization techniques previously considered for crop planning ranges from single to multiobjective and from linear to nonlinear forms, where computational intelligence techniques have been explored [18]. However, multiobjective optimization problems are frequently converted to single objective function optimization by means of weighting functions for several objective functions and solved using optimization techniques that are well suited for single objective function optimization. The relative importance of each objective function is expressed by the weighting functions. However, optimizing different objective functions concurrently without emphasizing the importance of each objective function a* priori* is called Pareto optimization. This relatively new optimization approach is more alluring for solving many nonlinear, multidimensional, multiobjective, combinatorial, nondifferentiable, nonconvex, and constrained practical problems often encountered in the real world phenomena.

## 2. Materials and Methods

### 2.1. Optimal Crop-Mix Planning Model

This section presents the mathematical formulation of the optimal crop-mix planning problem investigated in this work. The optimal crop-mix planning model is designed to maximize total net profit that can be produced by maximizing total crop production. The objective is to make an optimum use of the available limited resources in order to determine the land allocation for several competing crops required to be planted in a year. The soil characteristics, cropping patterns, crop produced, planting region, and cropping method are factors that contribute to the production cost, yield rate, and earning realized by a decision farmer. The crop-mix planning model is considered for a large scale planning incorporated with dataset collected from the South African abstract of agricultural statistics [19]. The model is specified as biobjective functions—profit function and crop production function with a set of constraints.

#### 2.1.1. Objective Function 1: Profit Maximization

Maximize

(1) F1=∑jm∑i∈Mj(Pi×Ui,j,k=1−B1)×Xi,j,k=1 +∑jm∑i∈Mj(Pi×Ui,j,k=2−B2)×Xi,j,k=2 +∑jq∑i∈Qj(Pi×Ui,j,k=3−B3)×Xi,j,k=3,

where

(2) $$\begin{array}{c} B_{s}=\left(V_{i,j,k=s}\times R_{i,j,k=s}\right)+F_{i,j,k=s}\;\forall s=1,2,3. \end{array}$$

*Objective Function 2: Crop Production Maximization*. Maximize

(3) F2=∑jm∑i∈MjGi,j,k=1×Xi,j,k=1 +∑jn∑i∈NjGi,j,k=2×Xi,j,k=2 +∑jq∑i∈QjGi,j,k=3×Xi,j,k=3.

The two objectives functions are to be concurrently solved, subject to the following constraints: food delivery, land allocation, labor cost, capital cost, and nonnegativity of decision variables.

*Food Delivery Constraint*. Consider

(4) ∑jm∑i∈MjGi,j,k=1×Xi,j,k=1  +∑jm∑i∈MjGi,j,k=2×Xi,j,k=2  +∑jm∑i∈MjGi,j,k=3×Xi,j,k=3≥Di ∀i.

*Land Allocation Constraint*. Consider

(5) $$\begin{array}{c} \sum_{i}\sum_{j}W_{k}\times X_{i,j,k}\leq L_{k},\;\forall k. \end{array}$$

*Labor Cost Constraint*. Consider

(6) ∑jm∑i∈MjTi,j,k=1×Xi,j,k=1  +∑jm∑i∈MjTi,j,k=2×Xi,j,k=2  +∑jm∑i∈MjTi,j,k=3×Xi,j,k=3≥Hk ∀k.

*Capital Cost Constraint*. Consider

(7) ∑jm∑i∈MjB1×Xi,j,k=1  +∑jm∑i∈MjB2×Xi,j,k=2  +∑jm∑i∈MjB3×Xi,j,k=3≤Ca.

*Nonnegativity Constraint*. Consider

(8) $$\begin{array}{c} X_{i,j,k}\geq 0\;\forall i,j,k, \end{array}$$

where

- *i* is a crop that can be considered for production,
- *j* is a crop combination made up of *i*,
- *k* is land type, *k* = 1 for a single-cropped land, *k* = 2 for a double-cropped land, and *k* = 3 for a triple-cropped land.
- *P*_*i*_ is price in South Africa Rand (ZAR) of crop *i* per metric ton,
- *V*_*i*,*j*,*k*_ is the variable cost required per unit area for crop *i* of crop combination *j* in land type *k*,
- *F*_*i*,*j*,*k*_ is the fixed cost required per unit area for crop *i* of crop combination *j* in land type *k*,
- *U*_*i*,*j*,*k*_ is number of farming units of crop *i* of crop combination *j* in land type *k*,
- *R*_*i*,*j*,*k*_ is cultivating land area ratio of crop *i* of crop combination *j* in land type *k*,
- *G*_*i*,*j*,*k*_ is yield rate, which is the amount of production in metric tons per hectare of crop *i* of crop combination *j* in land type *k*,
- *W*_*k*_ is land type coefficient for land type *k*,
- *D*_*i*_ is expected delivery (metric tons) of crop,
- *L*_*k*_ is available domain of land type *k*,
- *C*_*a*_ is working capital (ZAR), which indicates the total amount of money that can be invested for cropping,
- *M* is number of alternative crops for single-cropped land,
- *n* is number of crop combinations for double-cropped land,
- *q* is number of crop combinations for triple-cropped land,
- *M*_*j*_ is a crop in each *j* for single-cropped land, *j* = 1,…, *m*,
- *N*_*j*_ is the *j*th crop pair of the possible crop combinations of double-cropped land, *j* = 1,…, *n*,
- *Q*_*j*_ is the *j*th crop pair of the possible crop combinations of triple-cropped land, *j* = 1,…, *q*,
- *X*_*i*,*j*,*k*_ is the area of land in hectare to be cultivated for a crop *i* of crop combination *j* in land type *k*.

### 2.2. Multiobjective Metaheuristics

The multiobjective evolutionary metaheuristics are population based techniques for solving complex multiobjective optimization problems. A metaheuristic is an iterative master process that guides and modifies the operation of a subordinate heuristic to efficiently produce high quality solutions by exploring and exploiting a solution search space [20]. The synonymous underlying principle for all evolutionary metaheuristics is that, given an initial population of individuals, an environmental pressure causes the natural selection of the surviving individuals—leading to a rise in the population fitness. The most surviving individuals, according to their measures of fitness, would steadily progress to the next generation by the application of recombination and mutation operators that create diversity. The recombination operator is applicable to two or more selected parents to produce a set of offsprings. The mutation operator is applicable to a single parent to reproduce an offspring and selection operator guarantees the quality of individuals in the given population. The execution of recombination and mutation operators yields a set of offsprings that compete with the existing population members for a place in the next generation. This process is iterated to refine the individuals produced and the iteration comes to a stop when a quality individual is found or a given termination condition is satisfied.

In this study, we investigated a set of metaheuristics to test the performance of generalized differential evolution for multiobjective optimal crop-mix planning problem. These metaheuristics are *ε*-constrained, widely used in practice to solve multiobjective optimization; the nondominated sorting genetic algorithm (NSGA), one of the most popular elitist multiobjective evolutionary algorithms; and the generalized differential evolution 3 (GDE3), a more recent metaheuristic that finds a global optimum solution for a multiobjective optimization problem.

#### 2.2.1. Generalized Differential Evolution

The generalized differential evolution 3 (GDE3) [21] modifies the selection rule of the basic differential evolution (DE) [22] and extends DE/rand/1/bin strategy [23] to problems with *M* objective functions and *K* constraint functions. In DE/rand/1/bin notation, “rand” indicates how the vector for mutation is selected. The number of vector differences used in the mutation is indicated next, and “bin” indicates the way the old vector and the trial vector are recombined. The basic principle behind the selection rule is that the trial vector *u*_*i*,*G*_ is compared with the old vector *x*_*i*,*G*_. If the trial vector has an equal or lower objective value, then it replaces the old vector in the next generation [24]. This can be presented as follows:

(9) $$\begin{array}{c} x_{i,G+1}=\{\begin{array}{cc} u_{i,G} & \text{if}\;\;f\left(u_{i,G}\right)\leq f\left(x_{i,G}\right)\\ x_{i,G} & \text{otherwise}. \end{array} \end{array}$$

In the case of comparing feasible, incomparable, and nondominating solutions, both offspring and parent vectors are saved for the population of the next generation. This mechanism reduces the computational costs of the metaheuristic and improves its efficiency. The population size may increase at the end of a generation based on a similar selection method as used in NSGA-II; the population is reduced back to the original size. The sorting of the population members is based on the goal for a* posteriori* optimization. The worst population members are eliminated according to the principle of nondominance and crowding in order to reduce the population size to the original size. The GDE3 is similar to the differential evolution for multiobjective optimization (DEMO) [25], except that DEMO does not provide a mechanism for constraint handling nor recedes to basic DE in the case of a single objective. This is because DEMO modifies the basic DE and does not consider weak dominance in the selection. The GDE3 improves the ability to handle multiobjective optimization problems by giving a better distributed set of solutions and is less sensitive to the selection of control parameter values when compared to the earlier versions of GDE [26].

#### 2.2.2. The *ε*-Constrained

The *ε*-constrained optimization technique is capable of generating widespread alternative solutions to constrained multiobjective version of the crop-mix planning model. The *ε*-constrained technique is based on the principle of selecting one objective function—usually the most preferred one to be optimized—whilst the other objective functions are treated as constraints that are bounded by some allowable levels of epsilon, *ε*_*i*_ [27]. This implies that a single objective function optimization problem is defined for the most relevant objective functions subject to additional constraints. The levels of *ε*_*i*_ required to generate the entire Pareto optimal set for an optimization problem are then altered. The apparent difficulty encountered when using this technique is how to choose the *ε*_*i*_ values. It is practically hard to know beforehand what the best values will be. If the value of *ε* is slightly increased, it could lead to a lot of redundant runs and if the steps between different runs are too large, it is possible to miss Pareto optimal solutions.

#### 2.2.3. Nondominated Sorting Genetic Algorithm

The nondominated sorting genetic algorithm (NSGA) [28] has three essential properties: (i) it uses an elitist principle; (ii) it uses an explicit diversity preserving mechanism; and (iii) it emphasizes the nondominated solutions. In a typical scenario of the fast nondominated sorting, two entities are required to be computed: (i) domination count *n*_*p*_, which is the number of solutions that dominate the solution *p*, and (ii) *S*_*p*_, which is a set of solutions that *p* dominates. The solutions with *n*_*p*_ − 0 represent the first nondominated Pareto front. Thereafter, for each solution with *n*_*p*_ − 0, each member (*q*) of its set *S*_*p*_ is visited and its domination count is reduced by one to remove solution *p* from *n*_*q*_. The member for which the domination count becomes zero (*n*_*q*_ − 0) is put in a separate list *Q*, which represents the second domination front. These procedures are repeated for each member of *Q* to identify the third front and the procedure is continued until all fronts are identified. In order to obtain a density estimation of possible solutions surrounding a particular solution, the average distance of two points is computed on either side of the point along each of the objectives [29].

## 3. Results and Discussion

The multiobjective optimal crop-mix planning problem was solved using GDE3, NSGA-II, and *ε*-constrained. The population size was 100 and the number of generations was 50. The GDE3, NSGA-II, and *ε*-constrained metaheuristics were implemented using C-Sharp programming language in VISUAL-STUDIO version 2010 on an HP PC with Pentium dual core processor having 2.30 GHz clock speed and 4 GB of RAM. Simulation experiments were performed to determine the best values of step length “*F*” and crossover rate “CR” for better performance in GDE3 metaheuristic. The values of CR and *F* were varied from 0.1 to 1 with an increment of 0.1. The simulation experiments were conducted for each value of *F* with respect to all values of CR. Consequently, 100 such simulation experiments were performed.

The GDE3 was compared to NSGA-II and *ε*-constrained to investigate its performance when used to solve the multiobjective optimal crop-mix planning model considered in this work. It is interesting to discover that NSGA-II is very sensitive to the initial population. Due to the inability of NSGA-II to find a single feasible solution using many different seeds as experimented, the GDE3 was introduced to compare the outputs of the three metaheuristics. The improved selection based on crowding distance was demonstrated in the optimal crop-mix planning problem. The GDE3 found a solution that converged to the Pareto front in about 50 generations. In addition, the results of the comparative study show that better Pareto front is obtained by GDE3 with *F* = 0.5 and CR = 0.9. The control parameters for NSGA-II are the crossover probability *P*_*c*_ = 0.9 and mutation probability *P*_*m*_ = 1/*D* (*D* is the number of decision variables). The distribution index of crossover operator *η*_*c*_ = 20 and the distribution index of mutation operator *η*_*m*_ = 20. The number of the needed function evaluations for GDE3, NSGA-II, and *ε*-constrained was set at 10000.

In order to compare the performance of GDE3 with performances of NSGA-II and *ε*-constrained, 100 trial runs were conducted for solving the optimal crop-mix planning problem. The best front was obtained from GDE3 based on the statistical performance metrics of a*dditive epsilon indicator (AEI)*, g*enerational distance (GD)*, i*nverted generational distance (IGD)*, and s*pacing (S)* [30, 31].

Table 1 shows the results of AEI, wherein it can be seen that GDE3 recorded the best average value of 0.00824, closely followed by NSGA-II with a value of 0.00916, and *ε*-constrained ranked third with a value of 0.0109. The overall worst performing metaheuristic according to this metric is the *ε*-constrained.

Table 2 shows the results of GD, wherein it can be seen that GDE3 had the best performance, both in terms of the average value and the standard deviation, followed by NSGA-II. The GDE3 had the best average value of 0.00345, closely followed by NSGA-II with a value of 0.00406, and *ε*-constrained is ranked third with a value of 0.00412. The overall worst performing metaheuristic according to this metric is the *ε*-constrained.

Table 3 shows the results of IGD, wherein it can be seen that GDE3 gave the best result with an average value of 0.00281 followed by the NSGA-II with an average value of 0.00352. The NSGA-II and the *ε*-constrained were ranked second and third, respectively. The overall worst performing metaheuristic with respect to this performance metric is the *ε*-constrained.

Table 4 shows the result of spacing, wherein it can be seen that GDE3 gave the best result with an average value of 0.001469, closely followed by the NSGA-II with a value of 0.001534. The worst performing metaheuristic according to this metric is the *ε*-constrained.

In general, because performance metrics can sometimes be misleading in multiobjective optimization, it is always desirable to consider a graphical comparison whenever possible [32]. It is particularly germane when analyzing the Pareto front produced by a metaheuristic to consider two main evaluation criteria: (i) the placement of the solutions on the true Pareto front and (ii) uniform distribution of solutions along the Pareto front. These criteria were applied to establish graphical comparisons of the metaheuristics investigated in this work.

The first evaluation criterion requires us to determine the extent to which the points on the Pareto front are linearly correlated. Consequently, the metaheuristic that gives a Pareto front with points more linearly correlated is judged to be the best performing in solving the multiobjective optimal crop-mix planning problem. This approach is effective because it could indicate a natural association between crop production and net profit. The strength of the linearly of an association between two variables such as crop production and net profit can be determined by calculating the Pearson correlation coefficient. The correlation coefficient is a number between −1 and 1 that indicates the strength of the linear association between two variables, for instance, crop production and net profit. The higher positive value indicates a strong linear association in the same direction; that is, increase/decrease in one variable leads to increase/decrease in the other variable. If there is evidence of strong linearity, we would likely expect higher values of crop production to yield higher values of net profit. The second evaluation criterion suggests that solutions on the Pareto front should be uniformly distributed.

**Figure 1:**
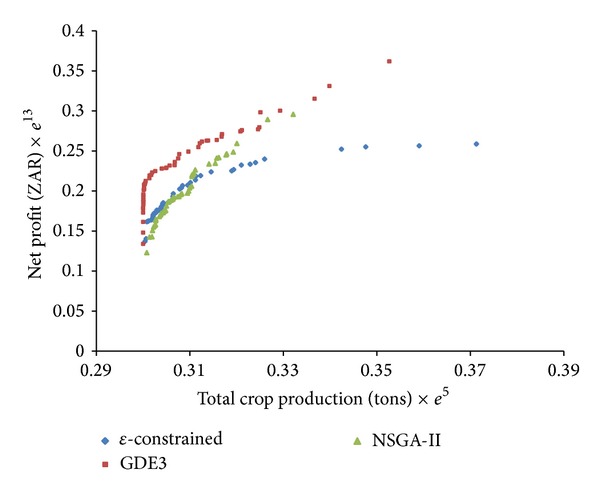
Pareto optimal set for GDE3, NSGA-II, and *ε*-constrained metaheuristics.

Figure 1 shows the Pareto fronts for the metaheuristics investigated in this work. In Figure 1, it can be seen that NSGA-II had a good distribution of solutions that it found, but it missed some portion of Pareto front. Hence, NSGA-II had an average performance of the multiobjective optimal crop-mix planning problem, despite its good spacing values. The correlation coefficient computed for the Pareto front of NSGA-II is 0.9711, which is slightly less than that of GDE3, which is 0.9736. The distribution of *ε*-constrained has better spacing but missed a considerable portion of the true Pareto front as in Figure 1. In addition, the metaheuristic has the least coefficient of correlation of 0.9708. It has been pointed out that having a good distribution of solutions becomes irrelevant when the metaheuristic does not converge to the true Pareto front of the optimal crop-mix planning problem [16]. The GDE3 as shown in Figure 1 clearly had the best overall performance, both in terms of distribution of solutions and the placement of solutions on Pareto front.

## 4. Implementation of a Crop Planning System

The crop planning system based on the generalized differential evolution 3 (GDE3) was implemented using the C-Sharp programming language in VISUAL-STUDIO. The purpose of the implementation was to provide a software tool to assist farmers in optimal crop planning decision making. The testing of the crop planning software was done with 10000 fitness function evaluations. The combination of parameters chosen for the testing is appropriate to have a reasonably good performance. This can be corroborated by checking the original sources of the GDE3. The varied operations such as capturing crop information and managing the information on crop combination as provided by the crop planning system could be used for crop planning related activities such as land allocation and crop selection. The recorded data are stored in a database for easy accessibility and could be used in various planning and decision making processes. The prototype system is relatively easy to use and simple to accommodate basic users with very little literacy levels.

**Figure 2:**
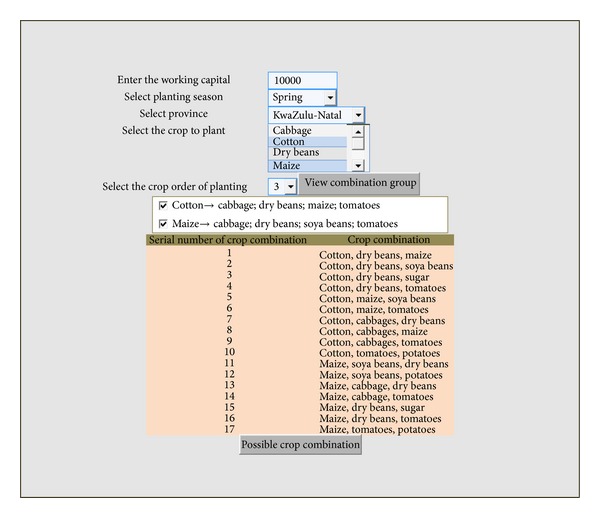
The screenshot of the process page of the decision support system.

The system was tested with a scenario where a household farmer has a working capital of R10,000 (unit in South Africa rand) with the land mass of 1 hectare. The farmer chooses to plant crops that could be planted along with cotton and maize such that the crop combination should be of order 3; that is the farmer decided to plant on a tricropped land. The farmer supplied all the necessary inputs and clicked on the button (view combination group) to view the crop combination group, consisting of crops that could be planted with the selected crops (cotton, maize). In order to view the number of possible crop combinations that could be obtained, the farmer selected any of the crop combination group of his/her choice and clicked the button (possible crop combination).  Figure 2 shows the screenshot of the process.

The system allocated a land portion to each crop combination; working with the scenario where the farmer decided to choose both combination groups, the system produced the result in Table 5. Finally, Table 6 shows the best result of the optimization process while maximizing total crop production and minimizing total planting area concurrently.

## 5. Conclusions

This work suggests that generalized differential evolution 3 (GDE3) is a useful multiobjective optimization tool for optimal crop-mix planning decision support. The metaheuristic is able to produce improved results when compared to those generated by other two metaheuristics that are representatives of the state-of-the-art in evolutionary multiobjective optimization. The GDE3 uses a simple mechanism to deal with constraints and the results computed by the metaheuristic generally indicate that such mechanism, despite its simplicity, is effective in practice.

The following conclusions can be made about the performance of GDE3: (i) GDE3 is able to produce most of the true Pareto fronts of the optimal crop-mix planning problem considered and it has the best performance; (ii) the GDE3 is able to produce a good distribution of solutions of the multiobjective optimal crop-mix planning problem; and (iii) GDE3 is ranked first with respect to the selected* performance metrics*. It can be concluded that GDE3 is practically effective for supporting optimal crop planning decision making process. Given the features of GDE3, an extension of the paradigm for multiobjective optimization can be particularly useful to deal with dynamic functions. The performance comparison of these metaheuristics is valuable for a decision maker to consider tradeoffs in accuracy versus complexity of solution techniques.

Future work will extend GDE3 for crop planning decision under uncertainty. This will produce a novel approach to deal with practical situations for which profit coefficients of agriculture are uncertain. The optimization approach can help farmers to efficiently utilize the available meager resources, including planting area, time, and money. The approach combines indigenous farming with information communication technology to optimize crop production, support efficient planning, and help farmers determine the possible combination of crops to plant on the same planting land year by year. As part of the future work, other optimization techniques can be compared to GDE3 to establish its superiority over many other techniques for crop planning decision making.

## Figures and Tables

**Table 1:** Additive epsilon indicator metric (10^−3^).

	GDE3	NSGA-II	ε-Constrained
Best	7.59	8.29	9.50
Average	8.24	9.16	10.90
Worst	9.65	10.5	13.10
Std. dev.	0.431	0.726	9.83

**Table 2:** Generational distance metric (10^−3^).

	GDE3	NSGA-II	ε-Constrained
Best	2.15	2.73	2.94
Average	3.45	4.06	4.12
Worst	6.87	7.10	7.92
Std. dev.	1.48	1.74	1.91

**Table 3:** Inverted generational distance metric (10^−3^).

	GDE3	NSGA-II	ε-Constrained
Best	2.40	2.62	2.73
Average	2.81	3.52	3.88
Worst	3.15	4.39	5.01
Std. dev.	0.171	0.398	0.512

**Table 4:** Spacing metric (10^−3^).

	GDE3	NSGA-II	ε-Constrained
Best	1.07	1.23	1.62
Average	1.47	1.53	1.92
Worst	1.69	2.03	2.84
Std. dev.	0.415	0.653	0.976

**Table 5:** The land allocation result.

Optimization		
Serial number of crop combination	Crop combination	Allocated land portion
1	Cotton, dry beans, maize	0.0538847660170879
2	Cotton, dry beans, soya beans	0.0552823360239594
3	Cotton, dry beans, sugar	0.0554001961353126
4	Cotton, dry beans, tomatoes	0.0537548193766635
5	Cotton, maize, soya beans	0.054378675341399
6	Cotton, maize, tomatoes	0.0537169683262593
7	Cotton, cabbages, dry beans	0.0560629255563752
8	Cotton, cabbages, maize	0.0529411764705882
9	Cotton, cabbages, tomatoes	0.0529411764705882
10	Cotton, tomatoes, potatoes	0.0530232065275474
11	Maize, soya beans, dry beans	0.053340093038395
12	Maize, soya beans, potatoes	0.054408535208579
13	Maize, cabbage, dry beans	0.0577712841409234
14	Maize, cabbage, tomatoes	0.0529411764705882
15	Maize, dry beans, sugar	0.0535396709191825
16	Maize, dry beans, tomatoes	0.0529411764705882
17	Maize, tomatoes, potatoes	0.0532893345994195

**Table 6:** Output of the optimization process.

Net profit (ZAR)	Total crop production (tons)	Total land utilization (ha)
**995.31296475439**	31.5857454386647	0.919617517093457
